# Impact of Drainage Parameters on Coal Fines Production in CBM Wells: A Long-Term Monitoring Study of Liulin Block, Ordos Basin, China

**DOI:** 10.1021/acsomega.4c10270

**Published:** 2025-02-17

**Authors:** Ziliang Liu, Yingchun Wei, Daiyong Cao, Zhenjiang You, Jin Zhang, Tao Meng, Anmin Wang, Yujie Yuan

**Affiliations:** † College of Geoscience and Surveying Engineering, China University of Mining and Technology-Beijing, Beijing 100083, China; ‡ State Key Laboratory for Fine Exploration and Intelligent Development of Coal Resources, China University of Mining and Technology-Beijing, Beijing 100083, China; § Centre for Sustainable Energy and Resources, Edith Cowan University, Joondalup, WA 6027, Australia; ∥ School of Petroleum, China University of Petroleum-Beijing at Karamay, Karamay 834000, China; ⊥ School of Engineering, Edith Cowan University, Joondalup, WA 6027, Australia

## Abstract

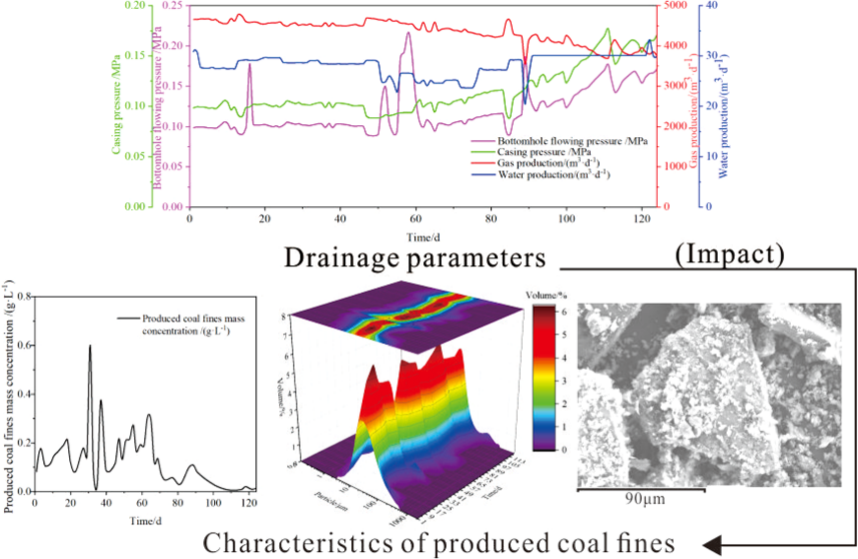

Coalbed methane (CBM) horizontal wells often experience significant coal fines production during the drainage process, which disrupts efficient CBM extraction. This study monitored key drainage parameters such as gas and water production rates, bottomhole flowing pressure, and casing pressure over an extended period. We analyzed fluid samples containing coal fines to understand their concentration, particle size distribution, and morphology. By correlating these findings with drainage parameters, we identified factors influencing coal fines production and the variation patterns in coal fines concentration. The results revealed that the concentration of produced coal fines ranged from 0.01 to 6.14 g/L, with particle sizes ranging from 0.63 to 704.00 μm. Rapid and continuous increases in production and gas release led to an increase in coal fine concentration and particle size. Conversely, slow, intermittent production increases and stable gas production helped reduce coal fines production. Additionally, pump start–stop cycles resulted in instantaneous increases in coal fines production. Notably, coal fines produced at the wellhead exhibited better roundness than those from workover operations. The smooth surface consists mainly of organic components, while the rough surface minerals are mainly kaolinite, calcite, quartz, illite, and pyrite. By advocating for a “continuous, slow, stable, and long-term” drainage approach, accidents such as screen clogging and pump blockage can be reduced.

## 1 Introduction

The issue of coal fines production during the development of CBM wells significantly impedes efficient CBM extraction.^1−4^ Coal fines production can clog the reservoir pore and fracture system, reducing reservoir permeability and impairing CBM well productivity.^5−8^ Coal fines can also cause drainage accidents such as borehole or screen plugging, stuck pump, and buried pump, resulting in discontinuous well drainage.^9−14^ Therefore, it is necessary to study the patterns and influencing factors of coal fines production from horizontal CBM wells.

Patterns of coal fine production in CBM development and the factors affecting coal fine generation have been investigated. These studies have employed on-site coal fines monitoring and laboratory testing methodologies.^15−18^ The causes of coal fine production have been categorized into geological and engineering factors based on examinations of coal fines. Previous studies have identified various influencing factors, such as hydrochemistry, coal components, coal structures, coal fines size, flow rate, surrounding pressure, etc.^19−28^ Throughout the development of CBM, the flow of fluids in the coal reservoir’s seepage channel initiates, migrates, *c* logs, and retains coal fines within the channel, supporting fractures, and at the well bottom. This process results in the continuous generation and migration of coal fines, leading to challenges such as buried pumps, stuck pumps, well workovers, and shutdowns when coal fines levels become excessive.

Geological factors primarily affect the generation of coal fines, but these factors remain consistent within a single well. As the CBM drainage process progresses, the dynamics of coal fines production are primarily governed by engineering factors, particularly changes in drainage parameters. This study specifically examines how variations in these drainage parameters impact coal fines production.

Previous researchers have monitored and analyzed the characteristics of coal fines produced by CBM wells, contributing valuable insights into their behavior and origins.^5,29−32^ For instance, Wei et al.^29^ studied the concentration, particle size, and morphological characteristics of coal fines in the Hancheng block of the Ordos Basin, identifying the coal structure as the primary controlling factor for coal fines production. Zhao et al.^30^ selected 12 CBM vertical wells with significant coal fines production in the Fanzhuang, East Qinnan, and Anze blocks of the southern Qinshui Basin, examining the effects of different production stages and coal structures on coal fines production. Zhang et al.^33^ focused on CBM wells at various drainage stages in the Linfen block, Ordos Basin, establishing quantitative relationships between coal fines concentration and influencing factors. Han et al.^34^ collected coal fines from CBM wells at different drainage stages in the Shizhuang block of the Qinshui Basin, studying the impact of geological factors on coal fines production. Most recently, Lyu et al.^35^ examined shallow CBM wells in the Panzhuang and Zhengzhuang blocks of the southern Qinshui Basin, along with deep CBM wells in the Wuxiang South block of the central and eastern Qinshui Basin, to study the characteristics of produced coal fines.

However, most of the existing studies have limited sampling durations or only analyze the characteristics of produced coal fines at different development stages, failing to reveal the dynamic and continuous coal fines production trends throughout the CBM development process. During the drainage process, fluctuations in parameters such as gas production, water production, casing pressure, and bottomhole flowing pressure can significantly impact coal fines production. Therefore, it is necessary to conduct the long-term monitoring of coal fine production in the same CBM well throughout various drainage and production stages, as well as during periods of well shutdown and workover. By integrating monitored drainage parameters, we can effectively investigate the influence of the CBM well drainage process on coal fines production.

In this study, long-term monitoring of drainage parameters, such as gas production, water production, bottomhole flow pressure, and casing pressure, was conducted in horizontal CBM wells. Moreover, coal fine-containing fluids were collected over an extended period. Methods including the mass conservation method, laser particle size tester, and SEM were employed to analyze the concentration, particle size, and morphology characteristics of the coal fines produced from horizontal wells within the Liulin block. By integrating the drainage parameters of CBM wells, we identified the influence of these parameters on coal fines production, uncovered the variation patterns in coal fines concentration, and proposed several control measures accordingly. The findings of this study contribute to establishing a theoretical framework for enhancing the efficiency and stability of CBM well production.

## 2 Geological Setting and Experimental Methods

### 2.1 Geological Setting

The Liulin block, the eastern margin of the Ordos Basin, encompasses geological features including the Lishi-Zhongyang syncline and the Wangjiahui anticline. The geological complexity is further accentuated by the presence of well-developed faults such as the Tanyaogou, Qingshanyuan, and Zhujiadian faults. It gradually transitions to a Liulin–Sanjiao monoclinic to the west. In the Liulin–Wubu area, the strata exhibit a gentle westward dip, forming a distinctive nose-like structure. Tensional forces along this axis have resulted in the formation of a near-EW-trending extensional fault zone, namely the graben composed of the north–south faults of Jucaita.^36^ The interior of the study area is a westward-dipping monoclinic structure with a stratigraphic dip angle of approximately 5°. Although large faults are relatively scarce in this area, the Jucaita fault and its associated smaller faults are prominent along the northern edge.

The primary coal seams targeted for CBM extraction include the No. 3 + 4, No. 5, and No. 8 + 9 seams. The CBM wells under our observation are mainly concentrated in the No. 3 + 4 and No. 5 coal seams. The No. 3 + 4 coal seams are located in the lower to middle sections of the Shanxi Formation. In some areas, it contains 1 to 3 layers of gangue. Overall, it is considered a stable and mineable coal seam. The No. 5 coal seam structure is relatively straightforward. In some areas, 1 and 5 layers of gangue are present in different parts. The seam is generally stable, allowing for efficient mining operations. The Jucaita Fault and its associated minor faults are prominent on the northern edge of the study area. Analyzing the logging data of the coal seams in the study area reveals that the northern coal seams are significantly affected by these faults, resulting in the widespread development of tectonic coal, primarily granulated and mylonitic coal. In contrast, the central part of the study area features a relatively simpler geological structure with the coal seams predominantly composed of primary structural coal and cataclastic coal. The six wells selected for this study are all located in the northern part of the study area, where tectonic coal is prevalent and the geological conditions are consistent. Specifically, wells LL-1 and LL-6 target the No.3 + 4 and No. 5 coal seams, while wells LL-2, 3, 4, and 5 focus on the No.3 + 4 coal seams.

### 2.2 Sample Collection

**Figure 1:**
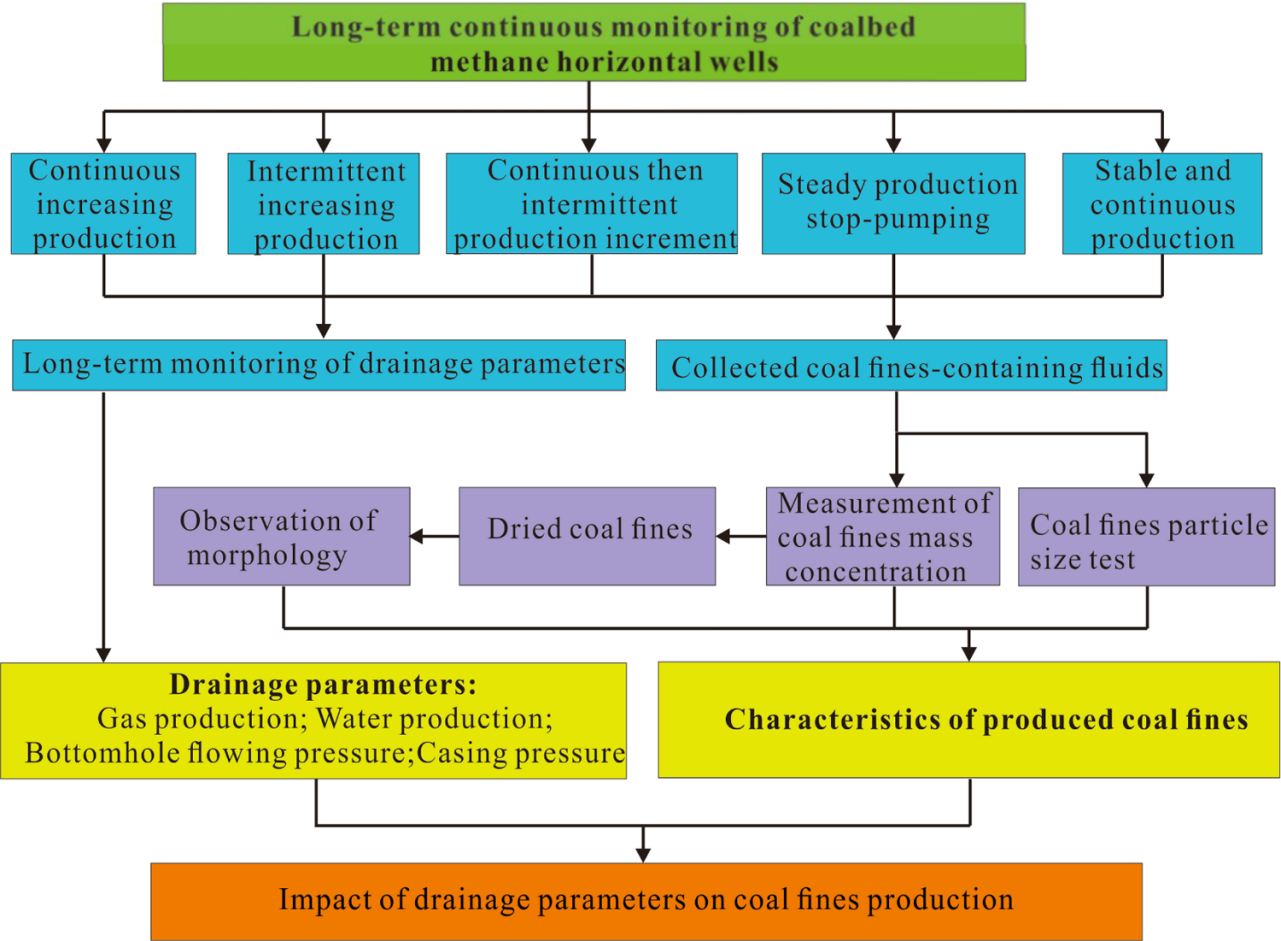
Flowchart of the experimental method.

To study the influence of various drainage factors on coal fines production during the drainage process of CBM horizontal wells, six wells known to experience coal fines problems were selected. These wells underwent continuous sampling of coal fines and monitoring of the CBM well drainage parameters. LL-1, 4, and 6 wells were monitored for 4 months, while LL-2, 3, and 5 wells were monitored for 8 months. Water samples containing coal fines were collected using a 0.5L vessel from the wellhead and during workover operations throughout the CBM drainage process (Figure 1).

### 2.3 Experimental Methods

The concentration, particle size, and morphology characteristics of the coal fines produced from horizontal wells within the Liulin block were analyzed (Figure 1).

(1) Measurement of coal fines mass concentration: First, the container containing coal fines liquid sample was thoroughly shaken. Subsequently, 100 mL of the sample was poured into a measuring cup, and filtration was performed using a vacuum pump (VP-2 rotary vane vacuum pump). For filtration, a hydrophilic double-sided organic PTFE microporous membrane was used. The pore size of the microporous membrane is 0.45 μm, and the tested coal fines have a minimum particle size of 0.63 μm, so it can be used. Before filtration, the membrane was dried at 40 °C for 2 h. The mass of the dried membrane was recorded.
  Following filtration, the filtered filter paper and coal fines were transferred to a vacuum drying box. The mixture was dried at 40 °C for 12 h, and the mass of the coal fines was then weighed and recorded. Subsequently, the drying process continued for an additional hour, and the mass was recorded again. This process was repeated until a constant mass of the filter membrane and coal fines was achieved. Finally, the mass concentration of coal fines was calculated based on the obtained measurements (Figure 1).
(2) Coal fines particle size test: The test was determined using a laser particle size analyzer (Microtrac S3500 & Bluewave). This analyzer operates based on the static laser diffraction method and utilizes the full-scale Mie theory. The test range is 0.01–2800 μm, and the measurement is conducted in a wet measurement, with pure water as serving the dispersing medium. The refractive index of the dispersing medium is 1.33, while that of the coal fines sample is 2.42.
(3) Observation of morphology: The S4800 cold field emission scanning electron microscope was employed. The coal fines were evenly spread and subsequently gold-plated. Images of the coal fines shape were captured in a secondary electron mode. The scanning electron microscope model S4800 boasts a resolution of 1.0 nm (15 kV) and 1.5 nm (5 kV), with a magnification range of 200–2000 times.

## 3 Experimental Results

### 3.1 Concentration of Produced Coal Fines and Drainage Parameters

The analysis of well drainage and the produced coal fine concentration revealed a range from 0.01 to 6.14 g/L. Under smooth drainage conditions, the coal fine concentration typically remains below 1 g/L. However, operational changes leading to increased production can trigger a sudden surge in coal fine concentration (Table 1).

**Table 1:** Monitoring Results of Coal Fines Concentration

well number	coal seams	production type	minimum value (g/L)	maximum value (g/L)	average value (g/L)
LL-1	3 + 4#, 5#	continuous increasing production	0.36	2.95	1.29
LL-2	3 + 4#	intermittent increasing production	0.01	6.14	0.47
LL-3	3 + 4#	continuous then intermittent increasing production	0.01	2.56	0.34
LL-4	3 + 4#	steady production stop-pumping	0.05	3.91	0.63
LL-5	3 + 4#	steady production stop-pumping	0.01	0.92	0.13
LL-6	3 + 4#, 5#	stable and continuous production	0.01	0.60	0.14

Based on the production behavior, they are classified into different types: continuous increasing production, intermittent increasing production, and stable production. While increased production operations generally elevate the produced coal fine concentration, the widening intervals between production increments moderately diminish coal fine production, consequently reducing concentration levels.

In the event of a pumping accident resulting in drainage cessation, coal fine concentration experiences a sudden surge upon pump restart. Conversely, CBM wells characterized by continuous and stable production maintain relatively low levels of coal fines concentration.

**Figure 2:**
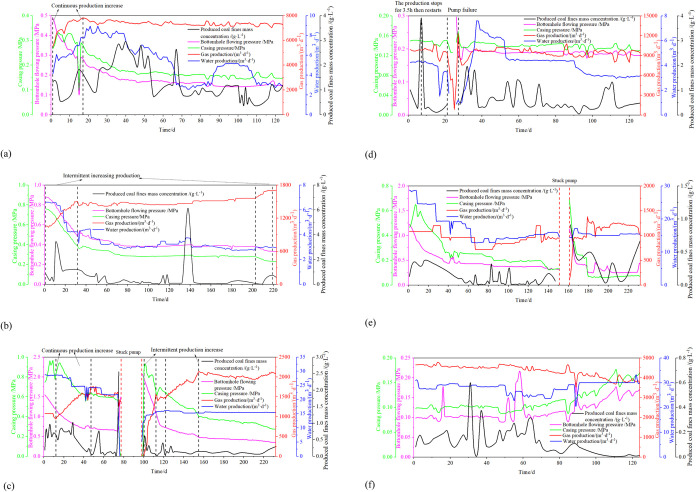
Drainage parameters of CBM horizontal wells and coal fines mass concentration: (a–f) LL-1–LL-6, respectively.

(1) Continuous increasing production type:
  The LL-1 well exhibited continuous production increases. The bottomhole flowing pressure at LL-1 CBM well decreased from 0.49 to 0.15 MPa, while casing pressure slowly decreased from 0.33 to 0.15 MPa. Water production varied widely, peaking at 8.94 m^3^/d and dropping to a minimum of 2.57 m^3^/d, while gas production initially rose from 5513 to 7,823 m^3^/d and then decreased, before stabilizing at approximately 7300 m^3^/d. The range of coal fines concentration was 0.36–2.95 g/L (Figure 2(a)). During a continuous production boost, gas production increased from 5,513 m^3^/d to 7,823 m^3^/d over 20 days, with coal fines concentration initially reducing to 0.55 g/L before rising to 1.82 g/L. This period saw fluctuations in bottomhole flowing pressure and casing pressure. As gas production progressed steadily, coal fines concentration was significantly affected by water production, with a similar fluctuation observed between the peak water production and coal fines concentration produced. Following 70 days of the gradual stabilization of gas and water production, the mass concentration of the produced coal fines gradually decreased and remained at 0.36–1.22 g/L, bottomhole flowing pressure slowly decreased to 0.15 MPa, casing pressure decreased to 0.15 MPa, and daily gas production decreased, before stabilizing at approximately 7300 m^3^/d.
(2) Intermittent increasing production type:
  LL-2 well adopts the intermittent increasing production method characterized by alternating production enhancement and stabilization phases. During this process, bottomhole flowing pressure and casing pressure drop gradually, while gas production rises steadily. The concentration of coal fines produced shows a tendency to increase in the early stage of production enhancement. However, effective stabilization of production can facilitate the discharge of coal fines from wells, ensuring a moderate level of coal fines production (Figure 2(b)).
  Over the monitoring period, bottomhole flowing pressure at LL-2 CBM well gradually reduced from 0.88 to 0.33 MPa, while casing pressure slowly dropped from 0.76 to 0.23 MPa. Water production decreased from 6.62 m^3^/d to 2.96 m^3^/d, while gas production gradually increased from 1032 to 1706 m^3^/d. The range of coal fines concentration was 0.01–6.14 g/L.
  Two intermittent production boosts were observed: the first occurred from day 4 to day 31, during which daily gas production increased from 1032 to 1503 m^3^/d. Coal fines concentration initially increased from 0.31 to 3.45 g/L and then decreased to 1.06 g/L. The bottomhole flowing pressure and casing pressure steadily decreased. In the early stage of production enhancement, the concentration of coal fines increased sharply. Due to the appropriate stabilization of production, coal fines produced by the perturbation of the coal seam were reduced and can be better discharged from the wellhead. The concentration of coal fines gradually decreased, reaching a stable level before the next production enhancement operation. The second increase in gas production occurred on day 203, rising from 1560 to 1706 m^3^/d, accompanied by an increase in coal fines concentration from 0.08 to 0.55 g/L.
(3) Continuous then intermittent increasing production type:
  The LL-3 well initially implemented continuous increasing production, resulting in significant drops in bottomhole flowing pressure and casing pressure alongside the rapid growth of gas production. Consequently, the concentration of coal fines increased sharply, impeding CBM well drainage and necessitating sand-fishing operations. Following the sand-fishing operation, an intermittent production increment method was adopted to restore production, leading to sustained lower levels of coal fines concentration (Figure 2(c)).
  Before the sand-fishing operation, LL-3 CBM well experienced a gradual decrease in bottomhole flowing pressure from 1.55 to 0.64 MPa, from 0.75 MPa gradually increased to 0.97 MPa, and then slowly decreased to 0.34 MPa. During this period, water production decreased from 28.73 to 9.19 m^3^/d, while gas production initially decreased from 1080 m^3^/d, gradually increased to 1759 m^3^/d, and then slowly decreased. The coal fines concentration was 2.56 g/L before well fishing and remained between 0.08 and 1.02 g/L for the rest of the time.
  Subsequent to the sand-fishing operation, intermittent production boosting was adopted, resulting in a gradual decrease in bottomhole flowing pressure from 2.09 to 0.35 MPa and a slow reduction in casing pressure from 0.93 to 0.27 MPa. Gas production rate gradually increased from 120 to 2,088 m^3^/d, while the concentration of coal fines ranged from 0.01 to 0.61 g/L (Figure 2(c)).
(4) Steady production stop-pumping type:
  Wells LL-4 and LL-5 operate under a steady production stop-pumping type, with both undergoing pump inspection operations. Following the well workover operations to stop discharge, there was a significant spike in coal fines concentration during the stop-pumping-start-pumping process (Figure 2(d,e)).
  On the 22nd day, the malfunction of LL-4 well prompted a check operation. Subsequently, the casing pressure, gas production, and water production decreased. The 28th day returned to normal, upon restarting the pump. Due to problems with the monitoring equipment, no bottomhole flowing pressure data was detected in the first 28 days. After the operation, the coal fines concentration surged from 0.42 to 1.96 g/L (Figure 2(d)).
  On the 151th day of sampling in LL-5 CBM well, the screw pump malfunctioned and pump checking operation was carried out. Before pump checking operation, the bottomhole flowing pressure gradually decreased from 1.23 to 0.32 MPa, while casing pressure slowly decreased from 0.65 to 0.13 MPa. Initially, water production decreased from 28.74 to 12.79 m^3^/d and then increased to 15.97 m^3^/d, while gas production initially decreased and then increased. The coal fines concentration ranged from 0.01 to 0.37 g/L. Following the pump-stopping operation, both casing pressure and bottomhole flowing pressure showed a decreasing trend, the coal fines concentration ranging from 0.03 to 0.92 g/L (Figure 2(e)).
(5) Stable and continuous production type:
  The LL-6 well operates under a stable and continuous production type, without undergoing gas production increase or pump checking operations. As a result, the drainage parameters have remained stable, maintaining the coal fines concentration is low. The continuous and stable production has effectively controlled the coal fines concentration (Figure 2(f)).
  The bottomhole flowing pressure fluctuated in the range of 0.1–0.22 MPa, while the range of casing pressure was 0.1–0.18 MPa. Water production averaged around 28.12 m^3^/d, with gas production averaging around 4,350 m^3^/d. The concentration of coal fines ranged from 0.01 to 0.60 g/L.

### 3.2 Particle Size of Produced Coal Fines

**Figure 3:**
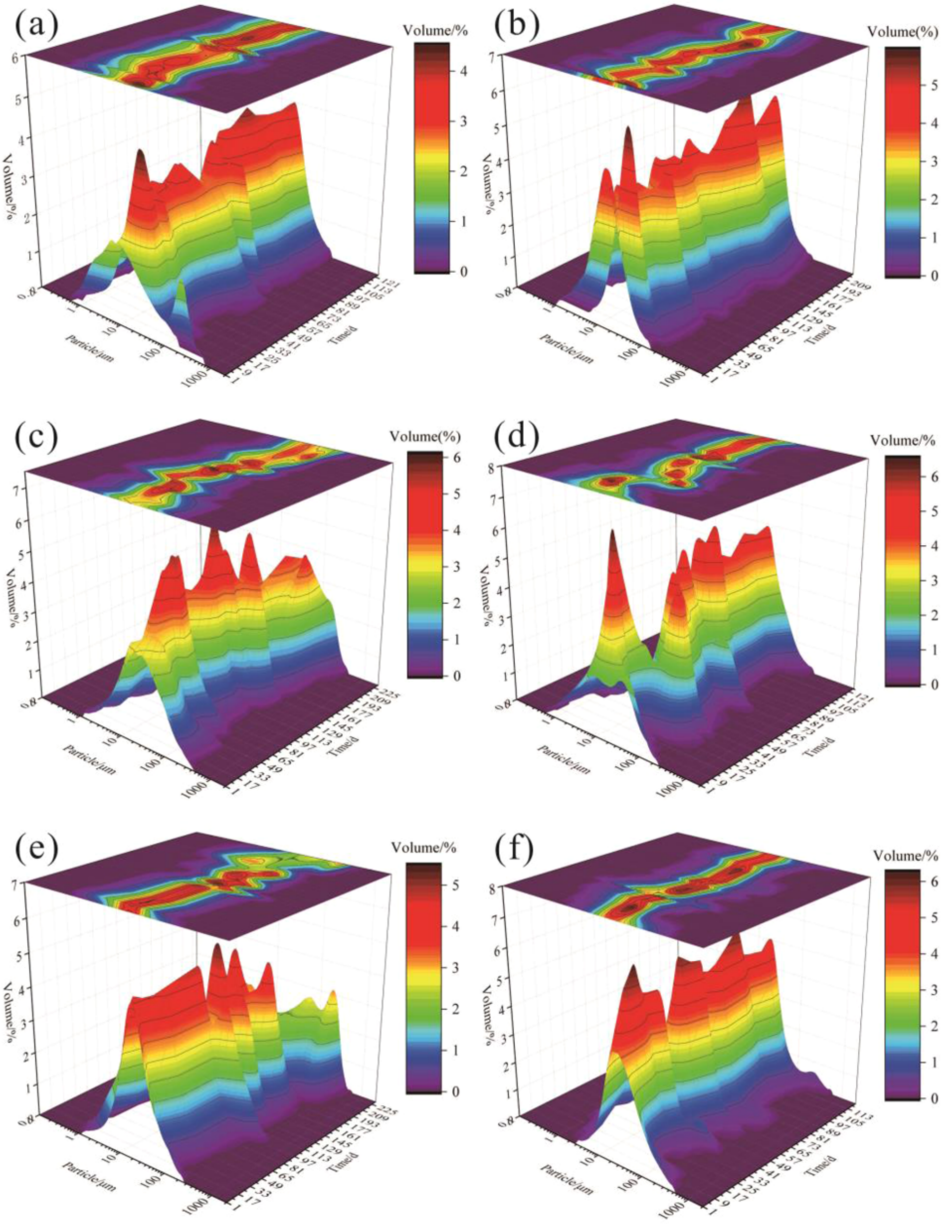
Produced coal fines particle size curve of different wells: (a)–(f) LL-1–LL-6, respectively.

The sizes of coal fines from horizontal wells ranged from 0.63 to 704.00 μm (Figure 3).

LL-1 CBM well is of continuously increasing production type. The particle size range of LL-1 CBM well produced coal fines is 0.63–704.00 μm. According to the drainage and production data, well LL-1 showed a dramatic increase in water and gas production from the very first day. The size distribution curve for coal fines in well LL-1 exhibited a bimodal distribution on the first day, while on subsequent days the distribution shifted to unimodal. Interestingly, the particle size of coal fines decreased on the fifth day, but with the continuous increasing production from the fifth to 15th day, the particle size of the produced coal fines steadily increased (Figure 3(a)).

The intermittent increasing production-type CBM wells LL-2 and LL-3 exhibited a particle size range for produced coal fines of 0.892–418.60 μm, with a particle size distribution curve displaying a unimodal distribution (Figure 3(b)). The LL-3 well size distribution curve transitions to bimodal only on the 197th day; otherwise, it exhibited a unimodal distribution, with subsequent size increase attributed to the increase in gas and water production (Figure 3(c)). These observations in wells LL-2 and LL-3 highlight the impact of intermittent increasing production operations on coal fines particle size (Figure 3(b,c)).

The steady production and pump-stopping-type CBM wells LL-4 and LL-5 produced coal fines with the size range of 0.63–352.00 and 0.75–592.00 μm. The malfunction of the LL-4 well on the 22nd day had prompted a check operation. LL-4 underwent a pump-stopping operation due to severe wear on the rotor of the screw pump caused by the increase in the frequency. Production resumed on the 28th day, leading to a shift in coal fines particle size distribution from a unimodal to a bimodal distribution (Figure 3(d)). In LL-5, following the pump stoppage and subsequent resumption of production, the particle size of coal fines increased to 592.00 μm, and the distribution curve shifted to a bimodal distribution (Figure 3(e)).

Well LL-6 operates as a stable and continuous production CBM well, yielding coal fines within a particle size range of 0.63–592.00 μm, with an average size range of 18.40–57.10 μm. The curves of coal fines from LL-6 exhibit unimodal distributions (Figure 3(f)). Throughout the monitoring period, there were no instances of production increase, and daily production volume remained relatively stable, resulting in minimal variations in coal fine particle size.

### 3.3 Morphology of Coal Fines

**Figure 4:**
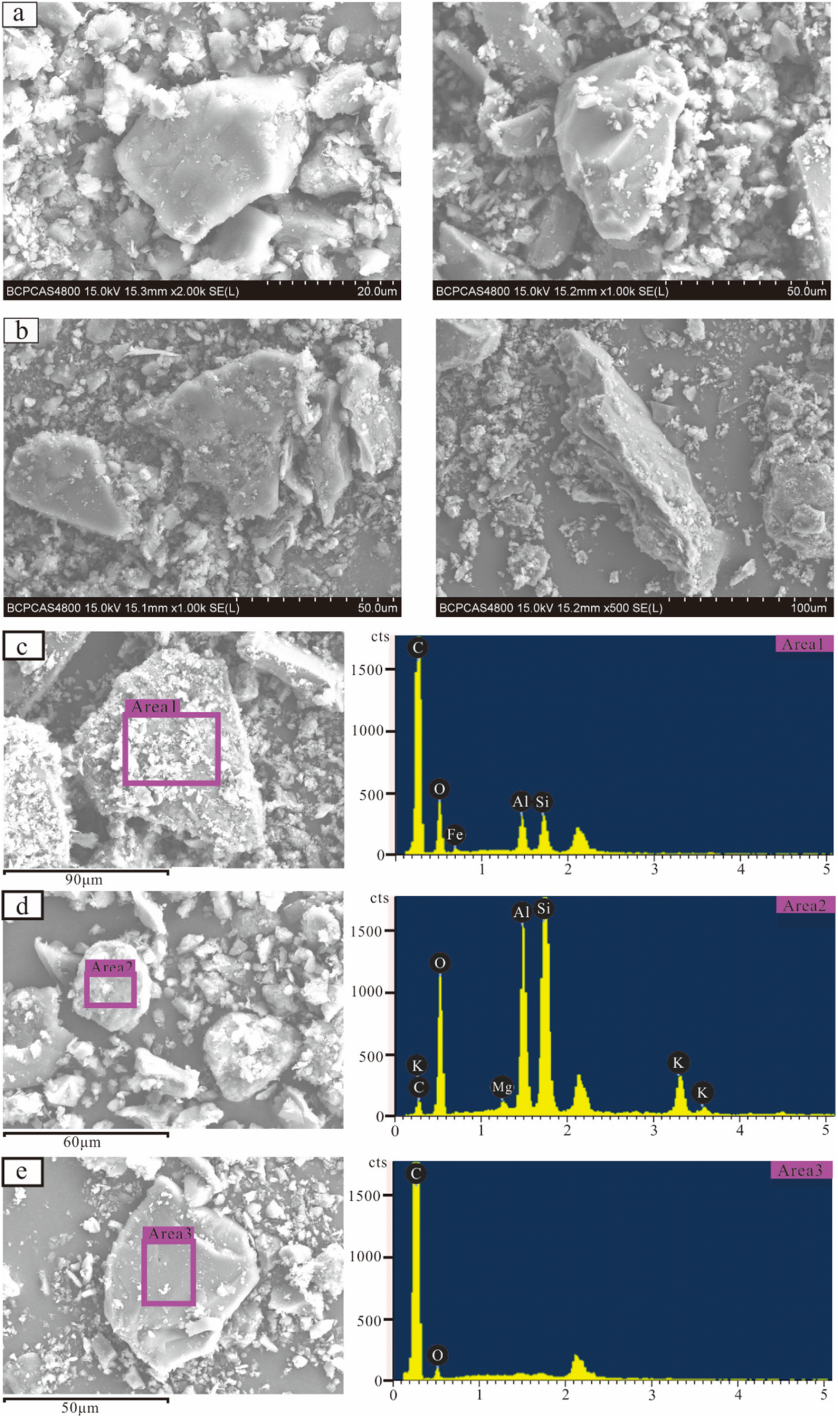
Morphology observation of produced coal fines: (a) produced from Well LL-1, Well LL-4wellhead; (b) produced from repair wells; (c, d) SEM image and EDS spectrum of rough surface; and (e) SEM image and EDS spectrum of smooth surface. Note that the unlabeled peak in each spectrum is due to Au, which was used for the sample coating.

The wellheads and repair wells that produced coal fines were subjected to morphological observation after filter-drying. Those from LL-1 and 4 wellheads exhibited predominantly spherical and ellipsoidal shapes, with less pronounced corners (Figure 4(a)). Conversely, the coal fines from repair wells appeared more massive, featuring angular roundness with more pronounced angles and inferior grinding roundness (Figure 4(b)). A comparative analysis of their morphology and surface characteristics indicated that coal fines from wellheads boasted superior roundness and smaller particle size compared to those from repair wells.

Coal fine surfaces can be categorized into two types based on smoothness: rough type and smooth type. Energy spectral tests were performed on both surface types. The rough-type surface of coal fines can be classified into two scenarios: mineral particles attached to the surface of coal fines (Figure 4(c)), with predominant elements being C, O, along with traces of Al, Si, and Fe, accounting for 73.48, 20.22, 2.3, 2.5, and 1.49% by mass, respectively. In contrast, numerous mineral particles enveloped the surface of coal fines or formed aggregates (Figure 4(d)). The main elements detected were C, O, Mg, Al, Si, K, with mass percentages of 12.33, 39.13, 0.65, 13.66, 27.69, and 6.54%, respectively. On smooth-type surfaces (Figure 4(e)), the predominant elements observed were C and O. The smooth surface is mainly composed of organic components, while the rough surface minerals are mainly kaolinite, calcite, quartz, illite, and pyrite.

## 4 Discussion

### 4.1 Relationship between Mass Concentration of Produced Coal Fines and Drainage Parameters

**Figure 5:**
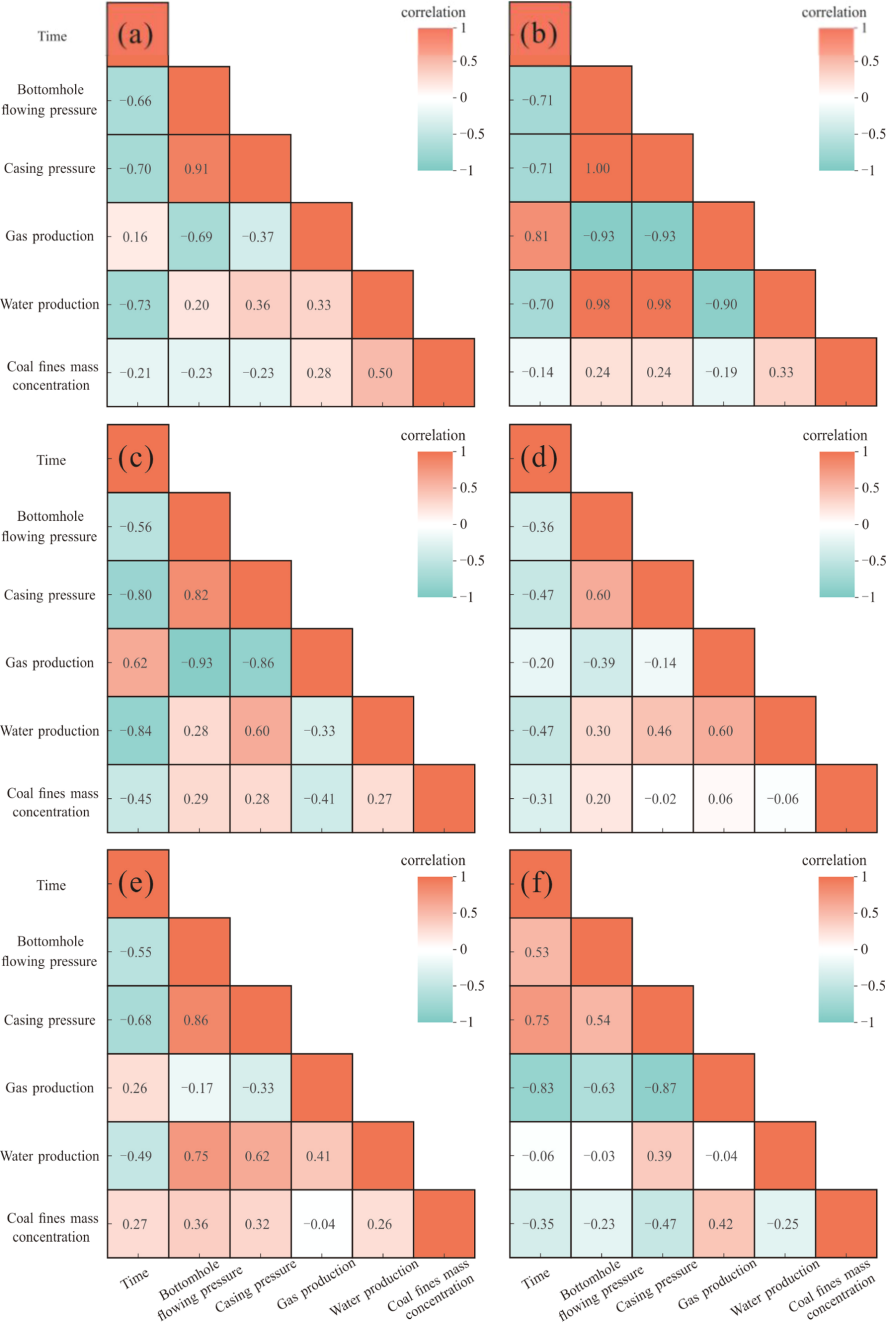
Correlation analysis between CBM well drainage parameters and mass concentration of produced coal fines: (a)–(f) LL-1–LL-6 respectively.

Along with the CBM drainage, factors including gas production, water production, bottomhole flowing pressure, casing pressure, and other operational conditions are interrelated and impact coal fines production. Due to the numerous factors affecting coal fines production, correlation analysis can effectively reveal the relationships between multiple variables, better demonstrating the correlation between different well drainage parameters and coal fines production Therefore, a correlation analysis was conducted on parameters such as gas production, water production, bottomhole flowing pressure, casing pressure, and produced coal fines mass concentration during CBM drainage (Figure 5).

Gas production stands as the most important parameter in CBM development, directly impacting the economic viability of the project. Observing the temporal evolution, we note that LL-1, 2, and 3 exhibit an upward trend in production, while LL-4 and 6 show a stable or slightly decreasing trend and LL-5 shows a steady or slightly increasing trend. Over time, water production demonstrates a negative correlation. Similarly, bottomhole flowing pressure and casing pressure exhibit negative correlations, except for the LL-6 well. Coal fines mass concentration shows a negative correlation except for the LL-5 well. These trends indicate that as CBM production proceeds and efforts are made to enhance production and conduct well repairs, coal fines production continues, leading to gradual reductions in bottomhole flowing pressure and casing pressure. This phenomenon may be attributed to coal fines aggregation causing blockages,^24,37^ thereby influencing fluid flow dynamics and trajectory curvature.^38^ The continuous coal fine production or the practice of temporarily stopping wells for repairs and subsequent discharge of coal fines effectively alleviates bottomhole flowing pressure and casing pressure, contributing to operational optimization.

In the correlation analysis between the mass concentration of produced coal fines and the CBM drainage parameters, it was observed that the correlation coefficients varied significantly across different wells. This variation indicates that a simple linear relationship is insufficient for comprehensively analyzing the production of coal fines. The variations across different wells are mainly reflected in their production types. Given the dynamic nature of drainage, it is essential to integrate diverse discharge profiles across different CBM wells. This holistic approach ensures a comprehensive consideration of how distinct well characteristics influence coal fines production.

### 4.2 Coal Fines Production Law and Influencing Factors in CBM Wells

The production enhancement method employed in CBM wells significantly affects the coal fine concentration (Figures 2 and 5).

Continuous increasing production-type well maintains stable daily gas production after the production enhancement, yet demonstrates a significant decline in bottomhole flowing pressure and casing pressure. The concentration of produced coal fines initially shows an upward trend following continuous production increase but decreases after a certain period (Figure 2(a)).

Intermittent increasing production-type well experiences gradual declines in bottomhole flowing pressure, casing pressure, and water production, coupled with a steady rise in gas production. Notably, coal fine production tends to increase in the early stage of the enhancement. However, maintaining a stable gas production rate helps reduce the coal fines production, keeping it at a lower level. When gas production is increased again, coal fines production rises accordingly (Figure 2(b)).

In the case of a continuous and then intermittent increasing production-type well, which initially adopts continuous production enhancement, substantial drops in bottomhole flowing pressure and casing pressure coincide with rapid gas production growth. At the same time, water production continued to decline. Consequently, there is a sharp increase in coal fine concentration, resulting in poor CBM well drainage and necessitating sand-fishing operations. Intermittent increasing production is employed to restore production following sand-fishing operations, resulting in a sustained low level of coal fines concentration. The bottomhole flowing pressure and casing pressure continued to decline, with little change in the water production (Figure 2(c)).

Both the wells with steady production stop-pumping experienced well shutdown and pump inspection operations. Upon pump restart, the fluid velocity surges, propelling a significant quantity of coal fines. Consequently, the coal fines concentration experiences a sudden spike postpump restart.^11^ The bottomhole flowing pressure and casing pressure exhibit either a decreasing or stable trend, with a noticeable decline following a fluctuation in water production (Figure 2(d,e)).

The stable and continuous production well has not undergone gas production increase or pump inspection operations and has maintained continuous and stable drainage. The variation in water production is minimal, while the bottomhole flowing pressure and casing pressure have slightly increased. Consequently, the concentration of coal fines has remained consistently low. This underscores how uninterrupted and stable drainage can effectively control the concentration of coal fines (Figure 2(f)).

The shrinkage effect was experienced by the coal matrix during the continuous desorption of CBM from its surface. Simultaneously, the generation of fractures and cleats, coupled with fluid action on the coal seam and drag force on the particles, leads to coal fines production.^33,39^ The smaller-sized fines, due to their low weight, readily suspend in flowing water, facilitating long-distance transportation.^40^ Abrupt fluctuations in bottomhole flowing pressure, casing pressure, gas production, and water production parameters can exacerbate this phenomenon, resulting in coal fines production with large particle sizes.^41^ Rapid decline of bottomhole flowing pressure can compress the coal rock matrix due to increased difference between the overburden and pore pressure, resulting in the erosion of coal reservoir by gas-liquid flows. Rapid changes in gas and water production accelerate the fluid flow in reservoir fractures, intensifying coal seam erosion.^42^ Therefore, rapid and continuous increases in production and venting activities result in the swift generation of coal fines, consequently elevating the concentration of coal fines.

Variations in the particle size of coal fines are intimately linked to fluctuations in gas and water production from CBM wells. Increased gas and water production, as well as the process of stopping and starting the pump, tend to result in larger particle sizes (Figures 2 and 3). The stable production-type CBM wells demonstrate minimal changes in coal fine size due to their consistent production operations. The smaller size of coal fines makes them easy to transport by flowing water, as they are lighter and can remain suspended in water for longer distances. As gas and water production increase, the higher flow rates of gas–water two-phase flow accelerate erosion of the coal seam, resulting in larger secondary coal fines generation. These larger particles are then transported out of the wellhead by the high-speed flow, causing the particle size of the produced coal fines to increase.^42−44^ After the pump was stopped, a significant accumulation of coal fines occurred in the fractures of the coal reservoir. Upon resuming discharge, the fluid velocity abruptly increases, causing a surge of larger particle size of coal fines into the pump barrel, and the produced coal fines particle size curve shifts to a bimodal distribution. Employing a slow and intermittent approach to increasing production and venting helps control coal fines generation. This gradual process, combined with the slow transportation of coal fines, ensures steady discharge from the wells and reduces the probability of clogging.^45,46^

There is a notable contrast in the morphological characteristics between coal fines produced during CBM well drainage and those from repair wells (Figure 4). The former typically comprises spherical and ellipsoidal coal fines with small particle sizes, facilitating their initiation, transportation, and discharge from the wellhead with fluids. Due to their long-distance transportation, the coal fines interact with the coal body, drainage equipment, and other particles, resulting in significant abrasion and rounding. Consequently, they exhibit rounded and subrounded shapes, with minimal edges and corners.^47^ In contrast, massive coal fines with larger particle sizes encountered in repair wells pose distinct challenges. They are less readily discharged from the wellhead and tend to accumulate at the bottom of the wellbore, potentially causing buried and stuck pumps. These fines are less involved in suspension and transportation processes, resulting in reduced friction between particles. When extracted during well repair, they typically exhibit a prismatic morphology with prominent edges and corners.^48,49^

### 4.3 Exploring Control Measures for Coal Fines in Horizontal CBM Wells

Since the commencement of CBM production in the Liulin block, instances of pump inspections due to pump blockages and screen tube clogging caused by coal fines have been prevalent, with pump inspections occurring on average every 6 months.

When coal fines enter the pump barrel, their concentration increases, leading to obstruction of the screen tubes and subsequent pump blockages. This results in elevated bottomhole flowing pressure, a drastic reduction in gas and water production, and a decline in pumping efficiency. Upon detecting a stuck pump, it is necessary to stop-pumping operations and conduct a thorough pump inspection, and upon resuming operations, daily water discharge can be restored to preoperation levels. However, gas production often fails to rebound to previous levels, exhibiting a downward trend once stabilized.^50,51^

The prevalence of screen pipe clogging and pump jamming incidents in CBM wells within the Liulin block can be largely attributed to the high coal fine concentration (Figure 2) and the presence of abundant clay minerals within the fines (Figure 4). Upon detecting coal fines clogging, the standard protocol involves halting well operations to initiate screen tube flushing. If the drainage performance remains insufficient even after flushing the screen tube, subsequent measures may include sand-fishing to address the issue effectively (Figure 2).

The rate of bottomhole flowing pressure reduction has a significant influence on CBM well productivity (Figures 2 and 5).^34,52^ Moreover, production-enhancing operations during the drainage process also greatly affect coal fines production. Thus, it is crucial to devise a comprehensive drainage system that addresses both the pressure reduction stage at the early stage of drainage and the subsequent gas rate rise stage during the production process.

During the initial drainage and pressure reduction stage, the primary focus is on the flowback of fracturing fluid by increasing the drainage rate to discharge the fracturing fluid and coal fines with smaller sizes out of the wellhead. However, an excessively rapid decline in pressure and a rapid increase in gas production impede reservoir permeability and the diffusion of the pressure drop funnel.^53−55^ Therefore, to maintain gas production efficiency, it is essential to regulate the drainage pressure decline rate at the early stage of drainage, ensuring controlled bottomhole flowing pressure reduction.

In the increasing stage of gas production, rapid and continuous increasing gas production exacerbates coal fine concentration, leading to screen pipe and well blockage, thus impeding drainage. Conversely, slow and intermittent increasing gas production mitigates coal fines accumulation, promoting stable wellbore discharge and reducing blockage incidents (Figure 2). Therefore, the CBM well drainage process should adhere to the principle of “continuous, slow, stable, long-term”, employing an intermittent production boosting approach of alternating between production increase and stabilization, so as to gradually increase gas production. This strategy effectively minimizes abrupt fluctuations in bottomhole flowing pressure and casing pressure as well as in gas and water production. Consequently, it reduces the generation of secondary coal fines and mitigates issues such as sieve tubing blockage and pump sticking, which are often caused by coal fines.^50,51,56−58^

## 5 Conclusions

In this study, long-term monitoring of drainage parameters and coal fines characters was conducted in horizontal CBM wells within the Liulin block. The influence of these parameters on coal fines production was identified, the variation patterns in coal fines concentration were uncovered, and several control measures were subsequently proposed.

(1) The concentration range of produced coal fines is 0.01–6.14 g/L. Rapid and continuous increases in production and gas release led to an increase in coal fines concentration. Conversely, slow and intermittent production increase coupled with stable gas production contributed to a decrease in the generation of coal fines. Furthermore, the cyclical starting and stopping of pumps caused abrupt surges in coal fines production.
(2) The size range of produced coal fines is 0.63–704.00 μm. After a sudden increase in gas and water production, the particle size increases significantly. Similarly, the particle size surges upon pump restart during production resumption. Conversely, CBM wells with stable production maintain consistent drainage, resulting in minimal variation in particle size.
(3) Coal fines collected at the wellhead exhibit higher roundness than those from workover wells. Smooth-surfaced coal fines primarily comprise organic components, while rough-surfaced ones contain minerals such as kaolinite, calcite, quartz, illite, and pyrite.
(4) Production enhancement operations during the drainage process significantly affect coal fines production. Adhering to the “continuous, slow, stable, and long-term” principle, efforts to minimize drastic fluctuations in drainage parameters are essential. Accidents such as screen clogging and pump blockage can be reduced.
